# Movement of Chikungunya Virus into the Western Hemisphere

**DOI:** 10.3201/eid2008.140333

**Published:** 2014-08

**Authors:** Roger S. Nasci

**Affiliations:** Centers for Disease Control and Prevention, Fort Collins, Colorado, USA

**Keywords:** Chikungunya virus, viruses, alphavirus, vectorborne, mosquito, Aedes aegypti, Ae. albopictus

Chikungunya virus (CHIKV) is an alphavirus transmitted in an urban epidemic cycle by the mosquitoes *Aedes aegypti* and *Ae. albopictus*. CHIKV outbreaks are characterized by rapid spread and infection rates as high as 75%; 72%–93% of infected persons become symptomatic. The disease manifests as acute fever and potentially debilitating polyarthralgia. In a variable proportion of cases, polyarthritis and fatigue can persist for 2 years or longer (*1*). During outbreaks, the large percentage of symptomatic infections places a considerable strain on resources of local health care providers and hospitals. Fortunately, death from chikungunya is rare.

CHIKV was first identified in Tanganyika (now Tanzania) in 1952 (*2*). The virus was later found to be widely distributed and to cause sporadic, mostly small outbreaks in Africa and Asia through the 1960s and 1970s. Little activity was reported from the mid-1980s until June 2004, when an epidemic occurred on Lamu Island, Kenya, then spread during 2005 to Comoros, La Reunion, and to other Indian Ocean islands, causing ≈500,000 cases (*3*). This was followed in 2006–2009 by an epidemic in India that produced >1.5 million cases in 17 of the country’s 28 states and subsequently spread through Southeast Asia to the islands of the Pacific Ocean (*4*). The public health community has come to recognize CHIKV as a major emerging, epidemic-prone pathogen.

The global expansion of CHIKV has been broadened by the movement of infected persons to areas with competent mosquito vectors and a susceptible human population (*5*). CHIKV-infected travelers have been documented in >22 countries throughout Asia, Europe, and North America (*1*,*6*,*7*); their travel led to outbreaks in northern Italy (*8*) and southern France (*9*). Until a few months ago, only travel-related cases had been detected in the Western Hemisphere (*7**,**10**,**11*) with no evidence of local transmission.

The first known autochthonous chikungunya cases in the Western Hemisphere occurred in October 2013 on the island of Saint Martin and were reported in December 2013 (*12*). During the next 4 months, >31,000 confirmed and probable autochthonous cases were reported from numerous other Caribbean islands (as of April 28, 2014: British territories Anguilla and British Virgin Islands; overseas departments of France consisting of Dominica, Guadeloupe, Martinique, Saint Barthélemy, and Saint Martin; constituent country of the Netherlands, Sint Maarten; the Federation of St. Kitts and Nevis; the Dominican Republic; and Saint Vincent and the Grenadines). Infected travelers originating from the island countries have carried the virus around the region, leading to authochthonous chikungunya cases occurring in mid-February 2014 in French Guiana on the mainland of South America. Virus spread to other island countries and expansion into mainland areas of South, Central, and North America are inevitable.

Three CHIKV genotypes (East-Central-South African [ECSA], West African, and Asian) have been described; apparently they evolved independently in the different regions (*13*). The ECSA genotype has primarily been associated with the current epidemics in the Indian Ocean region, and the Asian genotype has been associated with recent outbreaks in the Pacific region. A single-base mutation in 1 strain of the ECSA genotype enhances replication of the virus in *Ae. albopictus*, contributing to the explosive epidemic that was observed in the La Reunion outbreak (*14*). Enhanced *Ae. albopictus* competence is also produced by a different substitution in a CHIKV ESCA lineage that has been associated with an outbreak in Kerala, India, in 2009 (*15*). Sequence analysis demonstrated that an Asian genotype of CHIKV caused the current outbreak in the Caribbean (*12*). In this issue of Emerging Infectious Diseases (http://wwwnc.cdc.gov/eid/article/20/8/14-0333 ), Lanciotti and Valadere compare CHIKV strains circulating in the Caribbean to those obtained from human serum samples from locally transmitted cases on the British Virgin Islands in January 2014. Their findings indicate that the strain circulating in the Caribbean is most closely related to strains isolated in China during 2012 and from Yap, Federated States of Micronesia, during 2013–14 (*16*), confirming the extent and speed at which CHIKV strains move around the globe. 

Such knowledge about the specific virus lineage circulating in the region is essential to understanding the potential disease burden that may result. *Ae. aegypti* and *Ae. albopictus* are competent vectors of Asian genotype CHIKV (*17*), although there is little evidence supporting a substantive role of *Ae. albopictus* in epidemic transmission of the Asian CHIKV genotype. However, the capacity for *Ae. albopictus* to transmit Asian CHIKV provides the potential for introductions from the Caribbean islands, which will facilitate local transmission in areas of the continental United States and South America where *Ae. albopictus* is common, but *Ae. aegypti* is absent.

CHIKV has the same urban epidemic transmission ecology as dengue virus, with *Ae. aegypti* and *Ae. albopictus* serving as vectors (*6*). Like dengue, epidemic chikungunya is an anthroponosis that does not require a nonhuman vertebrate amplifier host. This means that the estimated 3.6 billion persons in 124 countries at risk for dengue (*18*) are at risk for chikungunya. In the Americas, dengue incidence has been increasing (*19*), indicating that the likelihood of CHIKV outbreaks is high in areas in the Americas where the population is prone to dengue. There are currently no CHIKV vaccines or specific treatments; the only public health intervention available is reduction of mosquito-to-human contact through personal protection measures and vector control efforts to reduce mosquito abundance.

The entry of CHIKV into the Americas was anticipated and prompted health agencies in the region to develop preparedness and response plans (*1*). Now that CHIKV is here, health agencies and health care providers in areas of the Americas where dengue is endemic, as well as in parts of temperate North and South America where *Ae. aegypti* and *Ae. albopictus* are present, should be aware of the potential for CHIKV introduction and establishment, particularly over the coming months as the rainy season starts and conditions that promote dengue transmission traditionally increase. Existing diagnostic and surveillance networks must be enhanced, and effective vector control activities must be intensified to address this new public health threat to the region.
